# Plants meet machines: Prospects in machine learning for plant biology

**DOI:** 10.1002/aps3.11371

**Published:** 2020-07-01

**Authors:** Pamela S. Soltis, Gil Nelson, Alina Zare, Emily K. Meineke

**Affiliations:** ^1^ Florida Museum of Natural History University of Florida Gainesville Florida 32611 USA; ^2^ Department of Electrical and Computer Engineering University of Florida Gainesville Florida 32611 USA; ^3^ Department of Entomology and Nematology University of California, Davis Davis California 95616 USA

Machine learning approaches are affecting all aspects of modern society, from autocorrect applications on cell phones to self‐driving cars to facial recognition, personalized medicine, and precision agriculture. Although machine learning has a long history, drastic improvements in these application areas recently have been driven by improvements to computational infrastructure; increased computing power; increased ability to collect, manage, and store very large amounts of data; and algorithmic advances. Multiple types of machine learning have been developed, each with its own techniques, strengths, and weaknesses, making certain approaches better matches for certain problems than others.

Supervised machine learning and the use of neural networks (e.g., deep learning; Table 1) underlie much of the recent accelerated application of machine learning to many biological problems, including those across a range of scientific questions in plant science. For example, deep learning technologies have recently achieved impressive performance on a variety of predictive tasks, such as species identification (Unger et al., 2016; Carranza‐Rojas et al., 2017), plant species distribution modeling (e.g., Zhang and Li, 2017; Botella et al., 2018), weed detection (Yu et al., 2019), and mercury damage to herbarium specimens (Schuettpelz et al., 2017). They are also being applied to questions of comparative genomics (e.g., Xu and Jackson, 2019) and gene expression (Mochida et al., 2018) and to conduct high‐throughput phenotyping (e.g., Singh et al., 2016; Ubbens and Stavness, 2017) for agricultural and ecological research. Moreover, novel approaches are poised to revolutionize studies of plant phenology (e.g., Pearson et al., 2020) and functional traits through application to more than 30 million images of herbarium specimens now available at iDigBio (http://www.idigbio.org) as well as other digital repositories.

**Table 1:** Glossary of terms related to machine learning.

Term	Definition
Artificial neural network	A type of machine learning algorithm whose computational model is (loosely) motivated by biological neural networks.
Deep learning	The use of artificial neural networks composed of many layers of neurons.
Supervised learning	A type of machine learning in which a model is fit using labeled training examples.
Unsupervised learning	A type of machine learning in which data samples are unlabeled. The goal of unsupervised learning is to uncover the latent structure in the data.
Clustering	A type of of unsupervised learning in which the goal is to partition the data into groups that are composed of similar samples.
Classification	A type of supervised learning in which the goal is to identify (i.e., classify) samples into one of several known categories.
Convolutional neural networks	A type of artificial neural network (or deep learning network, if the network consists of many layers) in which spatial arrangement of input data (e.g., pixels in an image) is leveraged during analysis.

The application of machine learning methods to extract data from herbarium specimens has grown and diversified in a few short years, beginning with species identification in a specific geographic region (e.g., Unger et al., 2016). Subsequent attempts to use deep learning to tackle the difficult taxonomic task of identifying species in large collections of herbarium specimens showed that convolutional neural networks trained on thousands of digitized herbarium sheets are able to learn highly discriminative patterns (e.g., Carranza‐Rojas et al., 2017). These results are very promising for extracting a broad range of accurate annotations in a fully automated way. Such approaches are also being applied to identification of plant phenophase (i.e., bud, flower, fruit), which is important for assessing the effects of climate change on plant growth and reproduction and for comparing plant responses with those of pollinators, migratory birds, and other species that rely on plants for food and/or nesting sites (see, e.g., Lorieul et al., 2019; Pearson et al., 2020; Brenskelle et al., 2020; Goëau et al., 2020). Likewise, other evolutionary or ecological traits, such as leaf shape and size, leaf margins, and flower color, could also potentially be scored from images of herbarium specimens. However, despite the promise of applying deep learning to herbarium specimen images to address a range of questions, this emerging field also raises challenging methodological questions about how to avoid any bias and misleading conclusions when analyzing the produced data. Indeed, as for any statistical learning method, convolutional neural networks are sensitive to bias issues, including the way in which the training data sets are built. Moreover, as good as the prediction might be on average, the quality of the produced annotations can be very heterogeneous from one sample to another, depending on various factors such as the morphology of the species, the storage conditions in which the specimen was preserved, and the age of the specimen when imaged. Given both the opportunities and challenges, additional research into the application of machine learning approaches to herbarium specimen images is needed to enable greater applicability to a broad range of scientific questions.

The field of machine learning is moving rapidly, with the development of alternative approaches that may be best suited to specific questions, data sources, and analytical techniques. This special collection of articles in *Applications in Plant Sciences* presents 16 papers, published across two issues of the journal, that explore methods and applications of machine learning to studies of plant ecology, morphology, genomics, and agriculture. The first issue comprises eight papers and focuses on applications to images of herbarium specimens, on topics from phenology to herbivory. The second issue includes papers that address a broader range of topics, data, and biological scale. We summarize the content of both issues here.

Plant phenological research has seen major advances in recent years through the use of herbarium specimens (Willis et al., 2017). Herbarium specimens collected over the past three centuries provide insight into flowering, leaf‐out, and fruit timing globally and across plant phylogeny (Davis et al., 2015). A major hurdle, however, is that to harness the full power of herbarium specimens for phenological research requires counting reproductive structures, which can be time consuming. Thus, automated recognition of reproductive structures on herbarium specimens is a key goal in current phenological research (Lorieul et al., 2019; Pearson et al., 2020). Two papers in this special issue address plant reproductive phenology. To make use of the extensive volume of herbarium specimens for examining angiosperm reproductive phenology, Goëau et al. (2020) applied a state‐of‐the‐art segmentation approach (*mask R‐CNN*) to automate locating, segmenting, and counting reproductive structures on images of herbarium specimens of *Streptanthus tortuosus* Kellogg (Brassicaceae). Phenological stages (i.e., buds, flowers, immature fruits, mature fruits) are distinct in *S. tortuosus*, and specimens were scored for phenophase. Evaluation of the performance of the method indicated that it shows particular promise in identifying the number of reproductive structures (accuracy was nearly 80%), but the accuracy of the results varied with respect to the training annotations, the type of reproductive structures scored, and the size of the reproductive structures. Although promising, these results suggest that further refinement is needed, and it is unclear how well the approach will scale to other species with different floral morphologies and perhaps less well‐differentiated phenophases.

To train machine learning algorithms to do this, however, will require massive input data to data‐hungry machine learning algorithms. In this issue, Brenskelle et al. (2020) assess the conditions needed for volunteers to help gather these data. The authors test for the effects of training type (in person or online), career stage, plant taxon, and phenological stage scored on the accuracy of volunteer‐provided phenological data from herbarium specimens. Regardless of expertise and training method, users provided highly accurate data, although data from people trained in person were more accurate than those trained online. This study provides a best practices guide for collecting annotation data. Importantly, the authors also demonstrate that online citizen science platforms might be able to provide accurate annotation data that can then be used downstream to train machine learning algorithms to recognize phenological stages.

Morphological variation, coupled with variation in the quality of herbarium specimens, leads to noise and potential bias in automated coding of characters from specimen images. Image segmentation is a computer vision algorithm that groups together pixels of an image that have similar attributes and generates a mask for each focal object in the image, such as a flower in an image of a herbarium specimen. Application of masks, such as those applied to plant phenophases, can help to reduce noise and bias. White et al. (2020) developed a workflow to apply segmentation masks to plant images using deep learning. Focusing on ferns, they generated a model that could segment herbarium images automatically, efficiently, and accurately across the morphological diversity of this clade. Although their study was restricted to ferns, the workflow is generalizable to all herbarium images and, with modification, may be applicable to other clades of plants with highly different morphologies.

Plants and insects have been interacting for 400 million years, and these interactions have likely driven diversification of both clades. The fossil record shows evidence of herbivory, providing a glimpse into long‐term patterns of plant–herbivore interactions and evolution. However, how herbivory changes over shorter timescales and geography is much less clear. Despite the fact that botanists generally attempt to collect specimens that are free of herbivore damage, herbarium specimens offer a view of plant–herbivore interactions over the past three or four centuries, with the potential to infer spatial and temporal patterns of herbivory, including response to climate change (Meineke and Davies, 2018; Meineke et al., 2018). However, manual scoring of insect damage to herbarium specimens is extremely laborious, and the possibility of applying machine learning to quantify the patterns and extent of insect damage to plant specimens is appealing. Meineke et al. (2020) initiated machine learning methods to explore their ability to classify multiple types of herbivory (and its absence) across a pair of divergent plant species. Although herbivory could not always be classified with high accuracy, the use of hand‐drawn boxes to locate areas of potential herbivory increased the accuracy of herbivory classification to 81.5%. The authors further identify ways to expand the accuracy of the models in future applications, potentially paving the way for exploring patterns of herbivory in relation to climate change, invasive species, and more.

The contributions of machine learning to the plant sciences, especially for automated species identification from images of digitized herbarium specimens, is showing great promise (Schuettpelz et al., 2017; Wäldchen and Mäder, 2018). This is especially true for genera with only slight morphological variation among species, particularly when compounded by hybridization and the presence of infraspecific taxa. Pryer et al. (2020) have built on this work with *Equisetum* L., a distinctive genus with 15 extant species complicated by morphological plasticity and frequent hybridization events that have resulted in a disproportionately high number of misidentified herbarium specimens. *Equisetum* includes two relatively distinct species (*E. hyemale* L. and *E. laevigatum* A. Braun) and a widespread, sexually sterile hybrid between them (*E. ×ferrissii* Clute) (Rutz and Farrar, 1984; Des Marais et al., 2003). The challenges faced here result from the cylindrical nature of the stem, which results in dramatic differences in specimen images due to factors such as the geometry of the flattened stems, the number of stems included on a single sheet, stem colors, and imaging parameters. Compounding the variations among images is the fact that accurate identification has more to do with the appearance of stem nodes and strobili than other features. Through successive testing of several models, Pryer and colleagues discovered that, out of 30 test images, 27 were classified correctly. Although the number of specimens is probably too small to be broadly generalizable, *E. hyemale* images were correctly classified in nine of 10 cases, *E. ×ferrissii* images in eight of 10 cases, and *E. laevigatum* images were never confused, resulting in an accuracy of 90%. These results suggest strong potential for machine learning’s impact on the accurate determination of closely similar taxa.

In their contribution, Ott et al. (2020) outline the development and output of GinJinn, object‐detection software designed to extract leaf images from herbarium specimens based on the TensorFlow (Abadi et al., 2016) object‐detection application programming interface (API), an API designed to make supervised deep learning object detection accessible for plant scientists. Although GinJinn makes heavy use of TensorFlow’s API, the authors maintain that GinJinn is not merely a wrapper for the API; it also provides data preprocessing, project set up, pretrained model download, simple model exporting, and the use of trained networks for the extraction of bounding boxes from newly acquired data. GinJinn was tested on a data set of 286 JPEG images of preserved plant herbarium specimens provided by the herbarium of the Botanic Garden and Botanical Museum Berlin‐Dahlem, Berlin, Germany. The images were annotated using the free open‐source tool LabelImg version 1.8.1 (https://github.com/tzutalin/labelImg), resulting in a total of 889 annotated intact leaves within 243 images of herbarium specimens of two species of *Leucanthemum* Mill. (the diploid *L. vulgare* Lam. and the tetraploid *L. ircutianum* DC.) known for their high variability in leaf shape. The task is complicated by the rare occurrence of intact leaves versus non‐intact leaves in these species. Using 183 specimens as the training data set, the GinJinn pipeline extracted one or more intact leaves in 95% of 61 test images.

A major challenge to cataloging and describing plant diversity lies in the development of high‐throughput technologies that facilitate rapid discovery of new taxa hidden in the backlog of still‐to‐be processed herbarium specimens. The 400,000 plant species currently known to science have required more than 250 years to name and classify, and as many as 70,000 flowering plant species are likely yet to be discovered (Joppa et al., 2011). Many of these may well be among the estimated one million specimens currently backlogged in herbaria. From Little et al.’s (2020) perspective, this renders herbaria largely untapped resources for the new and rapidly developing use of artificial intelligence (AI) in taxonomic research (Wäldchen and Mäder, 2018). To capitalize on this enthusiasm and encourage an increasing number of AI specialists to devote attention to algorithms that can produce species identifications, these authors mounted a Kaggle competition platform to crowdsource effective machine learning algorithms for analyzing plant specimen images. The competition data set included 46,469 images representing 683 species of the family Melastomataceae (Tan et al., 2019). In just two months, 254 models were developed that automatically identified the taxa among these digital representations, with the top four models identifying specimens to species with >88% accuracy.

Trait extraction from herbarium specimens can be laborious and time consuming, making the process an excellent candidate for the application of high‐throughput machine learning protocols and algorithms. Here, Weaver et al. (2020) describe and test LeafMachine, an automated, open‐source software tool for recognizing and measuring leaf dimensions from herbarium specimens and single leaf images across a wide range of largely woody taxa (trees, shrubs, lianas), although some herbaceous taxa were also included. The tests show varying results based on image resolution, specimen presentation, leaf condition, and whether leaf clumping was present. Of ~1000 images containing measurable leaves as confirmed through assessment, LeafMachine produced morphometric information for at least one leaf in 82.0% of high‐resolution images and 60.8% of low‐resolution images, suggesting positive results to the researchers but with a need for enhancement as machine learning technologies advance.

The second set of papers explores a broad range of topics, beginning with application of machine learning approaches to agriculture. The use of herbicides to control weeds in agricultural fields is costly both economically and environmentally, and alternatives are needed, especially for organic farming. Possible solutions include the use of targeted application of small doses of herbicide precisely on weeds via a robotic detector and application system and non‐herbicide methods of removal such as electrocution. However, such approaches to precision agriculture require highly accurate methods of detection and identification of weeds in agricultural fields. Champ et al. (2020) applied an instance segmentation convolutional neural network to robotically generated images of agricultural field plots to detect individual plants and then identify them as crops or weeds. Using this mask R‐CNN approach, the authors were able to correctly identify individual maize and bean crop plants at average precision values of 0.85 and 0.59, respectively; identification of weeds was generally more difficult, with average precision values as high as 0.73 for *Brassica nigra* W. D. J. Koch but less than 0.5 for the other weeds studied. Using these detection results, up to 60% of weeds could be removed, and plant centroids were more precisely located than with alternative bounding box approaches. Refinement of the models to account for plant species, plant size, plant position, and possible crop–weed interactions could improve accuracy for greater automated weed removal with fewer possibilities of confusion with crops.

Plant–insect interactions are biodiverse (Forister et al., 2015) and can be highly consequential for agricultural productivity (Sharma, 2014) and ecosystem function (Kurz et al., 2008). As a result, quantifying plant traits associated with resistance to insects is of broad interest in the natural sciences. One such type of defense against insect herbivores are trichomes, small hairs that serve as mechanical defenses that discourage insect herbivore feeding, oviposition, and movement. Like many such leaf traits, counting the trichomes required to address a given research hypothesis can be a Herculean task. Mirnezami et al. (2020) make advances toward automating quantification of trichome densities by capturing images of leaves, making the leaves transparent through a clearing process, and applying novel semi‐automatic and automatic methods for counting trichomes. They then compare results from these novel methods to manual counting and determine that the most accurate novel method was semi‐automatic (requiring input from the user) and was 90% accurate at estimating trichome densities on leaf surfaces. Although fully automated trichome counting has not yet been achieved, this study represents an important and detailed description of a major step forward in automated defense trait phenotyping for plants.

Given the ability to automate the estimation of plant traits, a follow‐on question would be whether the plant traits extracted could be reliably used for plant species identification. Furthermore, could the most informative traits for species identification be determined using machine learning approaches? Almeida et al. (2020) investigate the use of decision trees for plant identification using trait databases as well as identifying the most informative traits distinguishing between species. Using the TRY Plant Trait Database (Kattge et al., 2011, 2020) and a collection of species that spanned trees, herbs, grasses, and other taxa, they were able to correctly identify plant species with up to 90% accuracy in cross‐validation. Traits such as leaf shape, fruit type, and flower color were identified as being some of the most informative. As more plant trait data are collected (including by automated methods as mentioned above), the type of approach presented in this paper can be used to guide and inform the data collection process.

Acquiring high‐resolution images of plant root architecture for use in downstream analysis and machine learning algorithms has proved a challenging endeavor. Most current methods use techniques that are destructive to root architecture (e.g., Trachsel et al., 2011); involve ex situ imaging under controlled conditions, often using aboveground rhizotrons (chambers with windows into the soil of plants under cultivation); incorporate intrusive methods through which cameras are inserted into the ground (Johnson et al., 2001), sometimes by soil coring (Wu et al., 2018), with the tendency to disturb soil and roots; or use non‐intrusive methods such as ground‐penetrating radar for trees and woody plants with roots ≥1 cm in diameter or X‐ray computed tomography (Tabb et al., 2018) or magnetic resonance imaging (Pflugfelder et al., 2017) for pot‐grown plants with finer root systems. Ruiz‐Munoz et al. (2020) report on experiments to improve the resolution of these images by adapting two state‐of‐the‐art deep learning approaches, the Fast‐Super‐Resolution Convolutional Neural Network (FSRCNN) (Dong et al., 2016) and the Super Resolution Generative Adversarial Network (SRGAN). Their method is designed to estimate high‐resolution output from low‐resolution images to expose details not clearly delineated by a sensing device. Results of these evaluations demonstrate that these super‐resolution models outperform the basic bicubic interpolation even when trained with non‐root data sets.

Supervised machine learning methods are the methods most commonly used when applied to plant science. Often machine learning approaches are used to automate or reduce the effort and time needed to complete tasks that were traditionally completed manually by researchers. These sorts of tasks lend themselves well to supervised approaches. Yet, machine learning approaches also provide mechanisms for data mining and unsupervised exploration of collected data. Saryan et al. (2020) investigated and proposed the use of an unsupervised spectral clustering aid in discovery of species boundaries. The authors (with comparison to principal component analysis and non‐metric multidimensional scaling) determine that interactive spectral clustering can lead to improved partitioning and understanding in some problems and data sets.

Text recognition and mining are useful in a range of applications including the automated processing of specimen labels and search indexing. Thus, the automated recognition of Latin scientific names can be particularly useful for some applications. Little (2020) investigated and developed an open‐source browser‐executable approach for Latin scientific name recognition using artificial neural networks. The method relies on an ensemble network approach that can recognize Latin scientific names across a range of languages (e.g., Chinese, French, German, Japanese) with high recall and precision and at competitive speeds of 8.6 ms/word.

Plant genomes are generally large and complex, with multi‐gene families and high amounts of repeated sequences. With over 200 plant genomes now published (Chen et al., 2018), many more underway, and both genomic and transcriptomic resources available for thousands of other plant species (e.g., Matasci et al., 2014; Leebens‐Mack et al., 2019), data are now available for comparative analysis of plant genomes across phylogenetic scales. Although methods for identifying genic regions are currently quite successful, tools for inferring gene function and other attributes of plant genomes require further refinement. Machine learning approaches are being applied to a range of problems in plant genomics, and Mahood et al. (2020) review the promise of these methods. They focus on supervised machine learning for predicting gene function from sequence information as well as post‐genomic data. Because gene function may vary spatially and temporally within a plant and have either direct or indirect effects on phenotypes, functional prediction involves a combination of analyses aimed at genome structure, gene expression patterns, and protein–protein interactions, and the authors review machine learning methods aimed at each of these problems as well as those designed to integrate information across molecular and biological scales. Beyond introducing these methods, the authors identify current roadblocks to more efficient models and suggest possible solutions.

Many machine learning methods have been developed for visual imagery and text as outlined above. Yet more and more methods are being developed and adapted to non‐visual imagery such as X‐ray computed tomography, ground‐penetrating radar, and hyperspectral imagery (Zare and Ho, 2013; Rogers et al., 2016; Travassos et al., 2018). Théroux‐Rancourt et al. (2020) developed a three‐dimensional segmentation and characterization approach for leaf internal anatomy using X‐ray microcomputed tomography. The approach outlined by the authors leveraged a small number of hand‐segmented image slices to automate segmentation over more than 1000 scans with accuracies of greater than 90%. The approach is focused on segmented grapevine leaf scans while requiring minimal manual labeling, but highlights the possibilities of being able to apply machine learning methods to automate the analysis of a wide variety of data and image types.

The application of machine learning to questions in plant biology is still in its infancy, yet the promise of these methods to a broad range of problems is clear. From genomic tools to measures of plant morphology, growth, and development, and from assessing ecological interactions of plants with herbivores and their broader, changing environment to use in agriculture, new approaches involving machine learning have the potential to change how we study plants and even the questions we can ask. Further integration with fields ranging from subcellular to ecosystem scales, all likewise enabled by new machine learning approaches, will further enable new discoveries in plant biology. However, as the contributions to this special issue have cautioned, methods with sufficiently high accuracy for application are still under development and may require extensive investments in generating training data sets. Thus, despite the promise and appeal of machine learning approaches, certain problems may not be amenable either because of difficulty in refining the underlying model or because the data needed for appropriate training sets are not available or not easily acquired. We hope that the papers presented in this collection encourage further progress on the emerging applications of machine learning to plant biology.
